# The anticholinergic medication index and dementia risk: evidence from the UK Biobank and All of Us research program

**DOI:** 10.1093/ageing/afaf326

**Published:** 2025-11-06

**Authors:** Innocent Gerald Asiimwe, Kate Best, Reecha Sofat, Oliver M Todd, Lauren Walker, Andrea L Jorgensen, Andrew Clegg, Munir Pirmohamed

**Affiliations:** Department of Pharmacology and Therapeutics, Institute of Systems, Molecular and Integrative Biology, University of Liverpool, Liverpool, UK; Department of Health Data Science, Institute of Population Health Sciences, University of Liverpool, Liverpool, England, UK; Academic Unit for Ageing and Stroke Research, University of Leeds, Leeds, England, UK; Department of Pharmacology and Therapeutics, Institute of Systems, Molecular and Integrative Biology, University of Liverpool, Liverpool, UK; Academic Unit for Ageing and Stroke Research, University of Leeds, Leeds, England, UK; Academic Unit for Ageing and Stroke Research, Bradford Institute for Health Research, Bradford, England, UK; Department of Pharmacology and Therapeutics, Institute of Systems, Molecular and Integrative Biology, University of Liverpool, Liverpool, UK; Department of Health Data Science, Institute of Population Health Sciences, University of Liverpool, Liverpool, England, UK; Academic Unit for Ageing and Stroke Research, University of Leeds, Leeds, England, UK; Academic Unit for Ageing and Stroke Research, Bradford Institute for Health Research, Bradford, England, UK; Department of Pharmacology and Therapeutics, Institute of Systems, Molecular and Integrative Biology, University of Liverpool, Liverpool, UK

**Keywords:** ACMI, anticholinergic burden, All of Us Research Program, dementia, UK Biobank, older people

## Abstract

**Introduction:**

Anticholinergic drugs are associated with adverse effects, including cognitive decline. In this study, we externally validated the Anticholinergic Medication Index (ACMI) by investigating the association between baseline anticholinergic burden and dementia risk in two large prospective cohorts: the UK Biobank (UKB) and the US All of Us (AoU) program.

**Methods:**

We analysed data from the UKB (*n* = 125 260; study period, 2000–15) and AoU (92 047; 2000–22). Cox proportional hazards models, adjusted for clinical and genetic covariates with death as a competing risk, assessed the association between baseline annual ACMI-computed anticholinergic burden and dementia risk. Exploratory genetic analyses included candidate gene analysis of acetylcholine signalling pathway genes in the UKB and development of a polygenic hazard score in AoU.

**Results:**

Prescription of any of the 88 ACMI-listed drugs at baseline was associated with increased dementia risk (UKB HR: 1.15, 95% CI 1.09–1.21; AoU: 1.06, 1.04–1.09) and mortality (UKB: 1.23, 1.19–1.27; AoU: 1.16, 1.13–1.19). We replicated the genetic influence of *APOE* on dementia risk (UKB [*APOE ε4* vs *ε3* carriers] HR: 2.05, 1.86–2.26; AoU: 1.61, 1.44–1.80). No significant gene–drug interactions were identified.

**Conclusion:**

Higher baseline ACMI scores were associated with increased risks of dementia and mortality in two large cohorts, providing external validation of the ACMI. While causal inference is not possible, these findings support the ACMI’s potential utility as a prognostic tool for risk stratification and for informing future work on safer prescribing.

## Key Points

- Anticholinergic drugs are linked to adverse outcomes, including cognitive decline.
- Using the ACMI, a higher baseline anticholinergic burden was associated with increased dementia risk in two large prospective cohorts.
- Anticholinergic burden was also associated with increased mortality.
- *APOE ε4* strongly predicted dementia risk, but exploratory polygenic scores showed no interaction with anticholinergic burden.

## Introduction

Anticholinergic drug use, especially among older people with multimorbidity, has risen significantly [over nine-fold from 1990 to 2015 in UK Biobank (UKB) data], raising concerns about associated adverse effects including dementia [1–3]. This makes it clinically relevant to develop tools that can accurately estimate anticholinergic burden and predict related adverse effects, enabling optimised treatment for high-risk patients [4].

The Anticholinergic Medication Index (ACMI), a prognostic tool identifying patients at high risk of anticholinergic side effects like delirium or falls, was developed using data from 151 604 older people (65–95 years) in the ‘Connected Bradford’ database [5]. Among them, 31% were prescribed anticholinergic drugs, and 4% experienced delirium or fall-related hospitalisations. Patients on anticholinergic drugs had higher hospitalisation rates (4.8% vs 3.7%; *P* < .001). The ACMI showed strong predictive performance [5] and has been validated in the Secure Anonymised Information Linkage (SAIL) Databank.

Previous studies [2, 4, 6–8] have linked anticholinergic burden to dementia. Mur *et al*., using data from 171 775 UKB participants, reported that most anticholinergic scales predicted dementia [hazard ratio (HR): 1.03–1.13] with Durán *et al*.’s value-based scale showing the strongest association (1.12, 99% CI 1.04–1.22) [6, 9]. However, associations were largely driven by specific drug classes such as antidepressants, antiepileptics and antidiuretics. Unlike consensus-based scales such as the Anticholinergic Cognitive Burden (ACB) scale [10–12], which rely on simple point scores and have limited epidemiological validation, the ACMI was derived from prescribing data and validated against clinical outcomes of hospitalisation with delirium or falls [5]. Its design supports automated calculation within electronic health record (EHR) systems, enabling translation into routine care. Validating ACMI in large, independent prospective cohorts such as UKB and the US All of Us program (AoU) is therefore an important step to assess wider generalisability.

Building on previous research, we sought to validate the ACMI prospectively by investigating the association between baseline annual anticholinergic burden and dementia risk (with death as a competing risk) using UKB and AoU data. In addition, we conducted exploratory genetic analyses to assess whether variation in acetylcholine signalling pathway genes might modify these associations, using candidate gene analysis and a polygenic hazard score [13, 14].

## Methods

The reporting of this study follows the REporting of studies Conducted using Observational Routinely collected health Data (RECORD) statement [15] (Appendix Table S1) and the STrengthening the Reporting Of Pharmacogenetic Studies (STROPS) guideline [16] (Appendix Table S2).

### Data sources

We used two cohorts, the first being the UKB, a population-based prospective cohort of over 500 000 participants (aged 40–69 years at recruitment) recruited across the UK between 2006 and 2010 [17, 18]. Approximately 230 000 participants have linked health records, including death, cancer, hospital and primary care data. The UKB obtained ethics approval (11/NW/0382), and all participants provided written informed consent. Our study was approved under UKB application 56653.

The second cohort, the AoU Research Program, was launched by the US National Institutes of Health in 2018 to address diversity gaps in biomedical research and has enrolled over 700 000 participants, with 80% from underrepresented backgrounds [19]. Its repository, which includes EHRs and genomic data, is accessible through a cloud-based Researcher Workbench. Controlled-tier access, including genomic data, is granted to registered researchers who complete training and sign a data-use agreement [19, 20].

### Participants

Like Mur *et al*. [6], and considering the age-related increase in dementia risk, we included unrelated participants aged 60 years and over at dementia diagnosis or at the end of follow-up. Other inclusion criteria required no dementia diagnosis or prescriptions for cholinesterase inhibitors (donepezil, galantamine or rivastigmine) or memantine during year zero (the first year of registration or EHRs after 1999) or the following year, at least 2 years of registration records (year zero plus 1 year to rule out dementia diagnosis), and possessing genomic data. Participants with Parkinson’s, Huntington’s, Creutzfeldt–Jakob disease or multiple sclerosis at any point were excluded due to their increased dementia risk [6].

To develop (UKB) and test (AoU) a polygenic hazard score (PHS) for dementia, we restricted analysis to participants of European ancestry, as 96% of the UKB is White. Quality control excluded participants with non-European ancestry, discordant genetic and self-reported sex, sex chromosome aneuploidy, outlying heterozygosity, over 5% missing data or genetic relations to already included participants, as described previously [21].

### Outcome and follow-up

The outcome was the time to the first reported dementia diagnosis, with all-cause death as a competing risk. In the UKB, dementia cases were identified using UKB Category 1712 (‘Health-related outcomes first occurrences’), and Read and ICD-9/10 codes [6] from primary care data (Category 3000), hospital inpatient data (Category 2000), and death records (Fields 40001 and 40002), selecting the earliest complete record. In the AoU, standardised EHRs in the Observational Medical Outcomes Partnership (OMOP) Common Data Model were used to identify dementia cases from the ‘Condition’ or ‘Condition Occurrence’ table, with death recorded from the ‘Death’ table.

UKB prescription ascertainment is limited for years before 1999 [1, 6], so follow-up began on 1 January 2000 [or 1 year after General Practitioner (GP) registration for those registered after 1 January 1999; the first year of records was year zero]. For most participants (>70%, sourced from The Phoenix Partnership England), prescription records were available until 31 May 2016; therefore, prescriptions were considered until 31 May 2015 or 1 year before the latest records. In the AoU, follow-up also began on 1 January 2000 or 1 year after the first healthcare interaction (based on OMOP tables: ‘Condition Occurrence’, ‘Device Exposure’, ‘Drug Exposure’, ‘Measurement’, ‘Observation’, ‘Observation period’, ‘Procedure occurrence’ and ‘Visit occurrence’), with prescriptions considered until 1 July 2021, or 1 year before the latest records.

Primary care diagnosis records were available for most UKB participants until 31 May 2016 and hospital inpatient data until 31 October 2022. Using the later censor date would add 250 dementia cases but risk misclassifying one case as not dementia for every 3 additional cases (Appendix Figure S1). Therefore, we used the earlier censor date. For the All of Us program, Dataset v7 with a censor date of 1 July 2022 was used. Participants were followed until the first occurrence of dementia, death or the censor date and additionally, for the UKB, removal from a GP register without re-registration.

### Predictors

To estimate baseline anticholinergic burden during year zero (the first year of prescription records), we used the ACMI [5], which comprises 88 anticholinergic drugs (Appendix Table S3) and the Duran scale (147 drugs), previously identified as the top-performing scale [6, 9]. Mur *et al*. previously tested four scale ratings: a count-based (total annual number of anticholinergic drugs), value-based (count-based multiplied by the drugs’ anticholinergic values), dosage-adjusted (dose standardised by defined daily dose and multiplied by anticholinergic score) and quantity-adjusted (dosage-adjusted multiplied by drug quantity). They identified the value-based rating as the most strongly associated with dementia risk [6]. Accordingly, we used this rating and also included the simpler count-based rating for ease of implementation and interpretation.

Using UKB Resource 592 (‘Clinical coding classification systems and maps’), we mapped generic anticholinergic drug names to British National Formulary brand names and then to prescription records. Combination products were split into individual components for assessment. Topical, nasal, ear and eye preparations were excluded, except for certain transdermal patches (e.g. fentanyl, hyoscine) and inhalers (e.g. ipratropium, umeclidinium). In AoU, anticholinergic drugs were identified from the ‘Drug Exposure’ table.

Like Mur *et al*. [6], we included covariates such as age at index date, sex, data provider (UKB only), apolipoprotein E (*APOE*) carrier status (based on the SNPs rs429358 and rs7412) and comorbidities (depression, diabetes, hypercholesterolemia, hypertension and stroke) by year zero. We excluded covariates like education, socioeconomic status, body mass index, alcohol/smoking habits and physical activity because these were collected at UKB recruitment (2006–10), a median of 8.3 years (interquartile range: 6.7–9.4) after the start of follow-up (typically 1 January 2000, Appendix Figure S2), making them unreliable as baseline measures. Instead of including the index year as Mur *et al*. did, we used a binary variable (registration before vs. after 1 January 1999). Additional covariates included self-reported race, genotyping array (UKB only) and the first two principal components of genetic ancestry.

To explore genetic influences on anticholinergic effects and develop a polygenic hazard score, we analysed SNPs from 94 acetylcholine signalling pathway genes (Appendix Table S4) [22–25]. Genotyping, imputation and quality control for both cohorts are detailed elsewhere [18, 20], with additional filters applied for SNPs (minor allele frequency ≥ 1%, missing genotype rate < 5% and Hardy–Weinberg equilibrium *P* > 10^−6^).

### Sample size

We used the ‘ssizeEpiCont’ function in the R package powerSurvEpi [26] to calculate the minimum sample size for the primary analysis using pilot datasets of 1000 random participants from each cohort. Assuming 80% power, a significance threshold of 0.05 and a dementia hazard ratio of 1.12 [6], the minimum sample size was 60 727 for UKB and 20 316 for AoU.

### Missing data

In the AoU cohort, ~2% of the participants with missing/unusable sex information were excluded from analysis.

### Statistical analysis

We used Cox proportional competing risk models, adjusted for baseline annual anticholinergic burden and other covariates (see Predictors), with ‘dementia’ and ‘death without dementia’ as terminal states (Appendix Figure S3) [27]. Dementia cases diagnosed on the same day as death were treated as dementia cases. To compare the ACMI and Duran scales, as well as count-based and value-based ratings, anticholinergic burden was standardised to a mean of 0 and a standard deviation of 1. Results are presented as hazard ratios (HRs) with 95% confidence intervals (CIs).

Exploratory genetic analyses were performed in UKB and AoU, including candidate gene analysis of 94 acetylcholine signalling pathway genes (Appendix Table S4) and development of polygenic hazard scores. Full details of SNP selection, filtering, multiple testing correction and score derivation are provided in Appendix Text S1.

### Subgroup and sensitivity analysis

In the primary analysis, dementia was treated as an absorbing state (i.e. participants did not transition out of it). To account for deaths among dementia cases, we explored two multi-state models: one equating deaths with and without prior dementia (multi-state 1) and another distinguishing between them (multi-state 2, Appendix Figure S3) [27]. The proportional hazards assumption may not hold in all cases; when violated, we used the R flexsurv [28] package to conduct parametric transition-specific analyses. In the UKB, we used an earlier censor date (31 May 2016) to minimise misclassifying dementia cases, despite a shorter follow-up and reduced statistical power. To assess this impact, we performed a sensitivity analysis using the later censor date (31 October 2022). Additional analyses included a no competing risk model and exclusion of participants with an anticholinergic burden three or more standard deviations beyond the mean [6]. Lastly, to reduce protopathic bias, we excluded participants diagnosed with dementia within 1 year of follow-up—sensitivity analyses varied this cut-off from 2 to 16 years. All sensitivity analyses were conducted in the UKB.

## Results

### Participants

**Figure 1:**
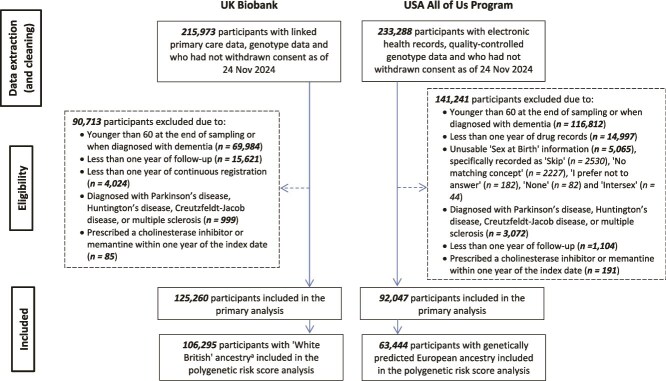
Flow chart for included participants. Bold values represent the total number of participants at each stage. ^a^This count excludes those who were additionally excluded due to discordant genetic and self-reported sex (*n* = 57), sex chromosome aneuploidy (*n* = 98) and being related to other included participants. (*n* = 3244).

Figure 1 shows the inclusion process for participants in the UKB and AoU cohorts for the primary (*n* = 125 260 vs. 92 047) and polygenic risk score (*n* = 106 295 vs. 63 444) analyses. Table 1 summarises participant characteristics. Compared to AoU, the UKB cohort had a lower median age at the start of follow-up (54.2 vs. 59.7 years) and a higher percentage of Whites (96.6% vs. 66.6%). Comorbidity profiles varied slightly, while *APOE ε4* carrier proportions were similar (~25%). Based on the ACMI, 24.6% of UKB and 22.3% of AoU participants used anticholinergic drugs, with usage ranging from 1 to 15 drugs in UKB and 1 to 22 in AoU. Dementia diagnosis rates were 1.5% (UKB) and 1.7% (AoU) after median follow-ups of 16.4 years (range 1.0–17.7) and 9.27 years (range 1.0–22.5). Appendix Tables S5 and S6 present the cohorts stratified by dementia status.

**Table 1:** Participant characteristics

Characteristic	UK Biobank (*N* = 125 260)	All of Us Program (*N* = 92 047)
**Age (years)**		
Mean (SD)	54.5 (5.87)	59.7 (9.69)
Median [Min, Max]	54.2 [42.8, 77.3]	59.7 [37.5, 115]
**Sex**		
Female	67 590 (54.0%)	51 071 (55.5%)
Male	57 670 (46.0%)	40 976 (44.5%)
**Race**		
White	121 028 (96.6%)	61 305 (66.6%)
Asian	2052 (1.6%)	1712 (1.9%)
Black	724 (0.6%)	15 491 (16.8%)
Mixed/other/unknown	1456 (1.2%)	13 539 (14.7%)
**Registration** ^**a**^ **after 1 Jan 1999**		
No	83 810 (66.9%)	12 692 (13.8%)
Yes	41 450 (33.1%)	79 355 (86.2%)
**Data provider**		
England (TPP)	88 412 (70.6%)	NA
England (Vision)	10 532 (8.4%)	NA
Scotland	13 594 (10.9%)	NA
Wales	12 722 (10.2%)	NA
**Genotyping array**		
Axiom	111 828 (89.3%)	NA
Bileve	13 432 (10.7%)	NA
**APOE carrier**		
ε2	16 278 (13.0%)	12 041 (13.1%)
ε3	77 154 (61.6%)	57 861 (62.9%)
ε4	31 828 (25.4%)	22 145 (24.1%)
**Prior depression**		
No	119 828 (95.7%)	87 248 (94.8%)
Yes	5432 (4.3%)	4799 (5.2%)
**Prior diabetes**		
No	119 511 (95.4%)	85 652 (93.1%)
Yes	5749 (4.6%)	6395 (6.9%)
**Prior hypercholesterolemia**		
No	115 478 (92.2%)	87 677 (95.3%)
Yes	9782 (7.8%)	4370 (4.7%)
**Prior hypertension**		
No	104 982 (83.8%)	75 253 (81.8%)
Yes	20 278 (16.2%)	16 794 (18.2%)
**Prior stroke**		
No	122 774 (98.0%)	92 029 (100.0%)
Yes	2486 (2.0%)	18 (0.0%)
**On at least one ACMI-listed drug**		
No	94 481 (75.4%)	71 504 (77.7%)
Yes	30 779 (24.6%)	20 543 (22.3%)
**ACMI (count-based scale)**		
Mean (SD)	0.406 (0.885)	0.638 (1.64)
Median [Min, Max]	0 [0, 15.0]	0 [0, 22.0]
**Dementia status**		
Censored	123 417 (98.5%)	90 483 (98.3%)
Got dementia	1843 (1.5%)	1564 (1.7%)
**Follow-up time, dementia (years)**		
Mean (SD)	13.8 (4.21)	10.8 (7.28)
Median [Min, Max]	16.4 [1.00, 17.7]	9.27 [1.00, 22.5]
**Death status**		
Censored	121 629 (97.1%)	90 773 (98.6%)
Died	3631 (2.9%)	1274 (1.4%)
**Follow-up time, death (years)**		
Mean (SD)	13.9 (4.15)	10.8 (7.30)
Median [Min, Max]	16.4 [1.00, 17.7]	9.31 [1.00, 22.5]

Abbreviations: ACMI, Anticholinergic Medication Index; APOE, Apolipoprotein E; Axiom, the Applied Biosystems UK Biobank Axiom Array; Bileve, the Applied Biosystems UK Biobank Lung Exome Variant Evaluation Axiom Array by Affymetrix; Max, Maximum; Min, Minimum; *N*, number of participants; NA, not applicable; SD, standard deviation; TPP, The Phoenix Partnership.

^a^For the All of Us Program, this is availability of drug records after 1 January 1999.

### Comparison of the Anticholinergic Medication Index and Duran scales in the UK Biobank cohort

**Figure 2:**
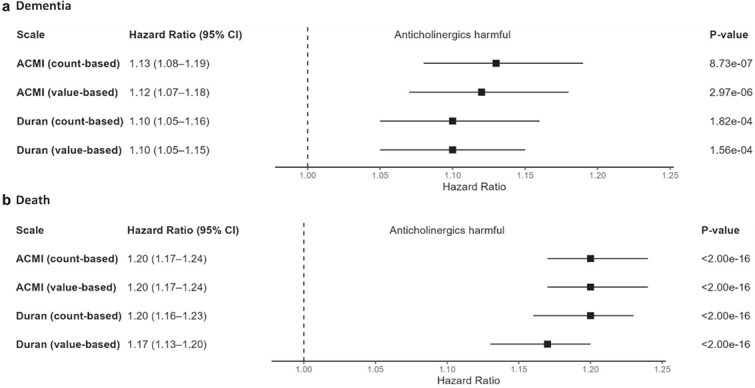
Hazard ratios for the association between anticholinergic burden and dementia (death as a competing risk) in the UK Biobank (a) or death (b). To enable a direct comparison between the different scales and scale ratings, the anticholinergic burden as computed by each scale/rating was scaled to have a mean of 0 and a standard deviation of 1. All models were adjusted for age at index date, sex, data provider, ‘registration before 1 January 1999’ status, race, genotyping array, apolipoprotein A carrier status, prior comorbidities (depression, diabetes, hypercholesterolemia, hypertension and stroke) and the first two principal components of genetic ancestry. ACMI, Anticholinergic Medication Index; CI, confidence interval.

In the UKB cohort, both the standardised ACMI and Duran scales showed significant associations with dementia and death risks (Figure 2). The ACMI count-based rating had the strongest association with dementia (HR: 1.13, 95% CI 1.08–1.19 per standard deviation increase in anticholinergic burden), performing better than the Duran scale (HRs: 1.10, 1.05–1.16), which was not further tested.

**Figure 3:**
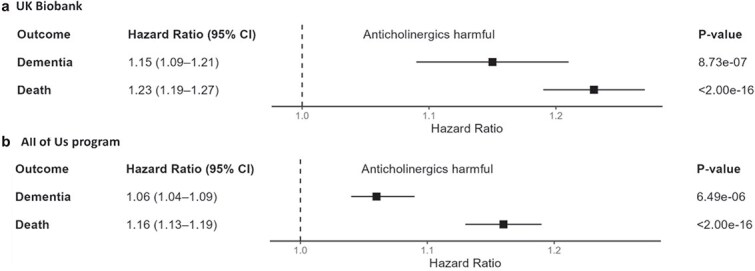
ACMI’s prediction of dementia (death as a competing risk) in the UK Biobank (a) and All of Us program (b). All models were adjusted for age at index date, sex, data provider, ‘registration before 1 January 1999’ status, race, genotyping array, apolipoprotein A carrier status, prior comorbidities (depression, diabetes, hypercholesterolemia, hypertension and stroke) and the first two principal components of genetic ancestry. ACMI, Anticholinergic Medication Index; CI, confidence interval.

Subsequent analyses used the ACMI count-based rating in its original form (i.e. not standardised to have a mean of 0 and standard deviation of 1). In its original form, the ACMI was associated with both dementia (HR: 1.15, 1.09–1.21 per additional anticholinergic drug) and death (HR: 1.23, 1.19–1.27; Table 2, Figure 3a). Subgroup analyses by race (Appendix Figure S4) confirmed a similar association in Whites (HR: 1.16, 1.09–1.22) but showed nonsignificant associations for the smaller race categories.

**Table 2:** Predictors of dementia (death as a competing risk) in the UK Biobank and All of Us program cohorts

Variable	UK Biobank (*N* = 125 260)				All of Us program (*N* = 92 047)			
	Dementia (events = 1843)		Death (events = 3631)		Dementia (events = 1564)		Death (events = 1274)	
	HR (95% CI)	*P*-value	HR (95% CI)	*P*-value	HR (95% CI)	*P*-value	HR (95% CI)	*P*-value
***Number of ACMI-listed anticholinergic drugs***	1.149 (1.087, 1.215)	8.73E-07	1.232 (1.191, 1.274)	<2.00E-16	1.065 (1.036, 1.095)	6.49E-06	1.163 (1.133, 1.194)	<2.00E-16
***Age (years)***	1.228 (1.216, 1.240)	<2.00E-16	1.157 (1.148, 1.166)	<2.00E-16	1.109 (1.103, 1.115)	<2.00E-16	1.084 (1.076, 1.091)	<2.00E-16
***Sex***								
Female	Reference							
Male	1.276 (1.163, 1.401)	2.63E-07	1.831 (1.708, 1.964)	<2.00E-16	0.957 (0.863, 1.061)	4.05E-01	1.572 (1.395, 1.772)	1.22E-13
***Race***								
White	Reference							
Asian	1.213 (0.494, 2.981)	6.74E-01	0.691 (0.388, 1.230)	2.09E-01	0.731 (0.391, 1.368)	3.27E-01	0.524 (0.233, 1.177)	1.18E-01
Black	0.475 (0.103, 2.180)	3.38E-01	0.298 (0.110, 0.809)	1.75E-02	0.760 (0.518, 1.114)	1.60E-01	1.165 (0.694, 1.957)	5.63E-01
Mixed/other/unknown	0.975 (0.537, 1.770)	9.35E-01	1.229 (0.837, 1.805)	2.92E-01	1.439 (1.199, 1.727)	9.17E-05	0.914 (0.711, 1.177)	4.87E-01
***Registered before 1 Jan 1999*** ^***a***^								
Yes	Reference							
No	0.481 (0.413, 0.560)	<2.00E-16	1.887 (1.742, 2.043)	<2.00E-16	1.403 (1.221, 1.612)	1.85E-06	3.699 (3.162, 4.329)	<2.00E-16
***Data provider*** ^***b***^								
England (TPP)	Reference							
England (Vision)	2.750 (2.401, 3.150)	<2.00E-16	0.003 (0.000, 0.021)	5.58E-09	NA	NA	NA	NA
Scotland	1.762 (1.537, 2.019)	4.09E-16	0.775 (0.695, 0.865)	5.73E-06				
Wales	3.020 (2.669, 3.418)	<2.00E-16	1.197 (1.074, 1.333)	1.09E-03				
***Genotyping array*** ^***b***^								
Axiom	Reference							
BiLEVE	1.060 (0.916, 1.226)	4.37E-01	1.261 (1.139, 1.395)	7.82E-06	NA	NA	NA	NA
***APOE carrier status***								
ε3	Reference							
ε2	0.900 (0.767, 1.057)	1.98E-01	0.994 (0.894, 1.105)	9.14E-01	1.007 (0.858, 1.183)	9.29E-01	0.912 (0.762, 1.091)	3.13E-01
ε4	2.054 (1.864, 2.263)	<2.00E-16	1.052 (0.972, 1.140)	2.09E-01	1.610 (1.439, 1.801)	<2.00E-16	1.072 (0.934, 1.233)	3.21E-01
***With prior depression***								
No	Reference							
Yes	1.273 (1.031, 1.573)	2.51E-02	1.107 (0.931, 1.315)	2.50E-01	1.970 (1.670, 2.323)	8.06E-16	1.380 (1.116, 1.705)	2.92E-03
***With prior diabetes***								
No	Reference							
Yes	1.016 (0.777, 1.328)	9.09E-01	1.493 (1.289, 1.729)	8.43E-08	1.503 (1.275, 1.773)	1.25E-06	2.060 (1.722, 2.465)	2.68E-15
***With prior hypercholesterolemia***								
No	Reference							
Yes	0.536 (0.419, 0.687)	8.17E-07	1.056 (0.922, 1.210)	4.31E-01	1.038 (0.873, 1.233)	6.75E-01	0.856 (0.694, 1.056)	1.47E-01
***With prior hypertension***								
No	Reference							
Yes	0.504 (0.427, 0.595)	5.85E-16	1.008 (0.900, 1.129)	8.87E-01	1.300 (1.148, 1.472)	3.47E-05	1.329 (1.139, 1.550)	3.06E-04
***With prior stroke*** ^***b***^								
No	Reference							
Yes	0.770 (0.515, 1.151)	2.03E-01	1.748 (1.445, 2.116)	9.45E-09	NA	NA	NA	NA
***PC 1***	1.001 (0.998, 1.005)	4.39E-01	1.002 (1.000, 1.004)	8.61E-02	0.454 (0.157, 1.315)	1.46E-01	0.694 (0.154, 3.127)	6.34E-01
***PC 2***	1.004 (0.998, 1.010)	1.91E-01	1.003 (0.999, 1.007)	9.25E-02	0.047 (0.007, 0.310)	1.51E-03	0.046 (0.003, 0.622)	2.05E-02

Abbreviations: ACMI, Anticholinergic Medication Index; APOE, Apolipoprotein E; Axiom, the Applied Biosystems UK Biobank Axiom Array; BiLEVE, the Applied Biosystems UK Biobank Lung Exome Variant Evaluation Axiom Array by Affymetrix; CI, confidence intervals; HR, hazards ratio; NA, not applicable; PC, principal component of genetic ancestry; TPP, The Phoenix Partnership.

^a^For the All of Us Program, this is first recorded electronic health record after 1 January 1999.

^b^Not included in the model for the All of Us program participants. For stroke, it was excluded since very few (<0.1%) participants had it.

Appendix Figures S5 and S6 present UKB sensitivity analyses, with results consistent with the primary findings. In Appendix Figure S6, increasing the cutoff beyond 1 year (primary analysis) reduced dementia cases, leading to wider confidence intervals and loss of significance at an 8-year cutoff. Appendix Figure S7 explains why a Gompertz distribution was chosen for the parametric transition-specific analyses.

### Performance of the Anticholinergic Medication Index in the All of Us cohort

In the AoU cohort, the ACMI (count-based) was associated with dementia (HR: 1.06, 1.04–1.09) and death (HR: 1.16, 1.13–1.19; Table 2, Figure 3b). Unlike the UKB, where significant associations were limited to Whites, AoU subgroup analyses showed significant associations in all racial categories except Asians for dementia (Appendix Figure S4).

### Exploratory genetic analyses

Exploratory genetic analyses in UKB (candidate gene analysis of 94 acetylcholine signalling pathway genes) and AoU (polygenic hazard scores based on UKB-derived effect sizes) did not identify significant gene–drug interactions after correction for multiple testing (Appendix Text S1, Appendix Tables S7, Appendix Figure S8). As expected, *APOE ε4* remained strongly associated with dementia risk (HR: 2.05, 95% CI 1.86–2.26, Table 2).

## Discussion

Using EHRs from the UKB and AoU cohorts, we found that prescribing any of the 88 ACMI-listed drugs was associated with increased dementia and mortality risks. While the link between anticholinergic burden and dementia is well established [2, 4, 6–8], our study further validates the ACMI for the outcomes of dementia and mortality. This demonstrates its robustness and generalisability in predicting dementia, highlighting its potential as a prognostic tool for clinical and research applications.

Compared to the Duran scale, previously identified as the best-performing scale in the UKB [6, 9], the ACMI showed a stronger association with dementia (HR: 1.13 vs. 1.10). While differences in methodology exist, the previously reported hazard ratios of the Duran scale (1.12 for both the count- and value-based ratings) are similar to our findings. Key differences include our exclusion of time-varying covariates, such as body mass index and alcohol/smoking status, which were collected during UKB recruitment but had a median gap of 8.3 years from our index date. We also included 1949 participants prescribed more than three ACMI-listed drugs, with sensitivity analysis showing consistent results (HR = 1.16 for three or less ACMI drugs versus 1.15 for the primary analysis).

Mur *et al*. explored alternative ratings, such as the quantity-based (HR = 1.12) and dosage-based (HR = 1.10), but these did not outperform count- and value-based ratings. Augmenting ratings with dose or quantity information might theoretically improve performance but is limited by inaccuracies in EHR data, particularly in capturing doses and quantities. Similarly, while value-based ratings theoretically add potency information, discrepancies in drug potency ratings across scales can undermine their effectiveness [9]. In our study, the simpler count-based rating slightly outperformed the value-based rating and was chosen for its ease of clinical interpretation, as hazard ratios reflect each additional anticholinergic drug.

Exploratory genetic analyses of 94 acetylcholine signalling pathway genes [22–25] did not identify significant associations after correction for multiple testing, consistent with the expectation of small effect sizes in gene–environment interaction studies [21]. Polygenic hazard scores in AoU, derived from UKB effect sizes, were also null. Nevertheless, our study replicated *APOE*’s role in dementia risk, an effect not observed for the death outcome (Table 2).

Our study had some limitations. UKB and AoU participants may not fully represent the general population, with UKB participants being healthier than average [29]. EHR data, not primarily collected for research [30], can be incomplete or inaccurate, may miss over-the-counter treatments and does not guarantee medication adherence. However, our use of a count-based rating mitigates some issues by not relying on precise dose or quantity data.

Dementia case identification in both UKB and AoU relied on EHR-based algorithms, which are prone to under-ascertainment and misclassification, as not all cases are clinically coded or captured in routine records. Diagnosis dates may also be imprecise if patients delay seeking care or remain asymptomatic. In the UKB, we excluded several potentially relevant time-varying covariates (e.g. BMI, smoking, alcohol use, physical activity and socioeconomic status) because they were collected a median of 8.3 years after baseline, which may have introduced residual confounding. This, however, is likely minimal given the similarity of our results to those of Mur *et al*. [6], who included these covariates. Although we adjusted for several relevant comorbidities at baseline, residual confounding by indication may remain, as some conditions leading to anticholinergic prescribing (e.g. depression, urinary incontinence, Parkinson’s disease) and their severity were not fully accounted for, and these conditions may themselves be associated with dementia risk or mortality.

Using baseline annual ACMI scores also does not reflect changes in prescribing during follow-up and may have caused exposure misclassification, although sensitivity analyses suggest our findings are robust. Moreover, the hazard ratios we observed were modest (HR ~1.06–1.15), and given the potential for residual confounding and exposure misclassification, these associations should be interpreted cautiously. Nevertheless, even small relative risks may have important population-level implications given the high prevalence of anticholinergic prescribing and the wide-ranging impact of dementia on individuals, families, carers and the health and social care system.

Finally, we did not assess individual drugs or classes, as our aim was to validate ACMI across two large cohorts; future work should address these effects, which may not be fully captured by weighted approaches. Despite limitations, our study’s strengths include the use of two large prospective cohorts and rigorous sensitivity analyses.

In conclusion, we found that higher baseline anticholinergic burden, measured using the ACMI score, was associated with increased risks of dementia and all-cause mortality in two large, independent, prospective cohort studies from the UK and USA. While these associations should not be interpreted as causal, our findings further validate ACMI for important outcomes and support its potential utility as a prognostic tool for risk stratification and guiding future research on safer prescribing.

## Supplementary Material

Supplementary_materials_afaf326

aa-25-2420-File003_afaf326

## Data Availability

The data that support the findings of this study are available from UK Biobank (https://www.ukbiobank.ac.uk/) and All of Us Research Program (https://allofus.nih.gov/), with the permission of UK Biobank and All of Us Research Program, respectively.
