# Supplementary material for: The anticholinergic medication index and dementia risk: evidence from the UK Biobank and All of Us research program
Source: Age Ageing. 2025 Nov 6;54(11):afaf326. doi: 10.1093/ageing/afaf326 (PMC12596250; doi:10.1093/ageing/afaf326)
Supplement: Supplementary_materials_afaf326 [file supplementary_materials_afaf326.docx]

# **The Anticholinergic Medication Index and Dementia Risk: Evidence from the UK Biobank and** **All of Us Research Program.**

**Table of contents**

**Supplementary Text…………………………………………………………………………………………….………….2**

**Supplementary Tables………………………………………………………………………………………….………….3**

**Supplementary Figures……………………………………………………………………………………………………12**

**Supplementary References..………………………………………………………………………………………..…20**

**Supplementary Text**

**Appendix Text S1.**

**Candidate gene analysis methods**

For the candidate gene analysis, each SNP in the 94 acetylcholine signalling pathway genes (Appendix Table S4), and its interaction with anticholinergic burden were added to the Cox proportional models using UKB data (*APOE* status was included as a covariate). To reduce the number of tested SNPs (*n*=29,775), we applied Haploview (v4.2) [1] with filters (-hwcutoff 0.000001 -tagrsqcutoff 0.8 -pairwiseTagging), retaining 7,562 SNPs. We controlled for multiple testing using the false discovery rate [2] with a significance threshold of 0.05. Effect sizes (log hazard ratios) and p-values were then used to generate polygenic hazard scores in the AoU cohort using the clumping (--clump-p1 1 --clump-r2 0.1 --clump-kb 250) and thresholding (p-values from 0.0001 to 1) technique in PLINK [3] as previously described [4]. Genomic coordinates were converted from the Genome Reference Consortium Human Build 37 (GRCh37, UKB) to GRCh38 (AoU), using the University of California (Santa Cruz)’s liftover tool [5]. Data preparation and other analyses were conducted using Python (v3.10) [6], Hail (v0.2.126, Apache Spark v3.3.0) [7, 8] and R (v4.3.2) [9].

***Candidate gene association analysis results in the UK Biobank cohort***

The most significant SNP was the cholinergic receptor muscarinic 3 (*CHRM3*) intron variant rs1594513 (SNP:AMCI HR 1.19, 1.09–1.29, *P* = 4.49E-05) but its FDR-corrected *P*-value (0.270) exceeded the 0.05 threshold (Appendix Table S7). *APOE* *ε4* carriers had a strong association with dementia (HR: 2.05, 1.86–2.26, ­*P* < 2.00E-16, Table 2), with this effect not seen with death (HR: 1.05, 0.97–1.14, *P* = 0.209).

***Development of a*** ***Polygenic Hazard Score (PHS) in the All of Us cohort***

Using the effect sizes and p-values from the UKB candidate gene analysis, we tested several p-value thresholds in the AoU cohort (63,444 participants of European ancestry), but none were significant at the 0.05 threshold (Appendix Figure S8).

**Supplementary Tables**

**Appendix Table S1. The RECORD statement – checklist of items, extended from the STROBE statement, that should be reported in observational studies using routinely collected health data.**

|  | Item No. | STROBE items | Location in manuscript where items are reported | RECORD items | Location in manuscript where items are reported |
| --- | --- | --- | --- | --- | --- |
| Title and abstract | 1 | (a) Indicate the study’s design with a commonly used term in the title or the abstract (b) Provide in the abstract an  informative and balanced summary of what was done and what was found | Title and abstract | RECORD 1.1: The type of data used should be specified in the title or abstract. When possible, the name of the databases used should be included.  RECORD 1.2: If applicable, the geographic region and timeframe within which the study took place should be reported in the title or abstract.  RECORD 1.3: If linkage between databases was conducted for the study, this should be clearly stated in the title or abstract. | Title and abstract |
| Introduction | | | | | |
| Background/rationale | 2 | Explain the scientific background and rationale for the investigation being reported. | Introduction |  |  |
| Objectives | 3 | State specific objectives, including any prespecified hypotheses. | Introduction |  |  |
| Methods | | | | | |
| Study design | 4 | Present key elements of study design early in the paper. | Data Sources |  |  |
| Setting | 5 | Describe the setting, locations, and relevant dates, including periods of recruitment, exposure, follow-up, and data collection. | Data Sources |  |  |
| Participants | 6 | Cohort study – Give the eligibility criteria, and the sources and methods of selection of participants. Describe  methods of follow-up. | Data Sources, Participants | RECORD 6.1: The methods of study population selection (such as codes or algorithms used to identify subjects) should be listed in detail. If this is not possible, an explanation should be provided.  RECORD 6.2: Any validation studies of the codes or algorithms used to select the population should be referenced. If validation was conducted for this study and not published elsewhere, detailed methods and results should be provided.  RECORD 6.3: If the study involved linkage of databases, consider use of a flow diagram or other graphical display to demonstrate the data linkage process, including the number of individuals with linked data at each stage. | Data Sources, Participants |
| Variables | 7 | Clearly define all outcomes, exposures, predictors, potential confounders, and effect modifiers. Give diagnostic  criteria, if applicable. | Outcome and Follow-up, Predictors | RECORD 7.1: A complete list of codes and algorithms used to classify exposures, outcomes, confounders, and effect modifiers should be provided. If these cannot be reported, an explanation should be provided. | Outcome and Follow-up, Predictors |
| Data sources/ measurement | 8 | For each variable of interest, give sources of data and details of methods of assessment (measure-ment). Describe comparability of assessment methods if there is more than one group. | Outcome and Follow-up, Predictors |  |  |

**Appendix Table S1. Continued.**

|  | Item No. | STROBE items | Location in manuscript where items are reported | RECORD items | Location in manuscript where items are reported |
| --- | --- | --- | --- | --- | --- |
| Bias | 9 | Describe any efforts to address potential sources of bias. | Statistical Analysis |  |  |
| Study size | 10 | Explain how the study size was arrived at. | Sample Size |  |  |
| Quantitative variables | 11 | Explain how quantitative variables were handled in the analyses. If applicable, describe which groupings were chosen, and why. | Statistical Analysis |  |  |
| Statistical  methods | 12 | (a) Describe all statistical methods, includ ing those used to control for confounding  (b) Describe any methods used to examine subgroups and interactions.  (c) Explain how missing data were addressed.  (d) Cohort study - If applicable, explain how loss to follow-up was addressed.  (e) Describe any sensitivity analyses. | Statistical Analysis |  |  |
| Data access and cleaning methods |  | .. |  | RECORD 12.1: Authors should describe the extent to which the investigators had access to the database population used to create the study  population.  RECORD 12.2: Authors should provide information on the data cleaning methods used in the study. | Statistical Analysis |
| Linkage |  | .. |  | RECORD 12.3: State whether the study included person-level, institutional-level, or other data linkage across two or more databases. The methods of linkage and methods of linkage quality evaluation should be provided. | Data Sources |
| Results | | | | | |
| Participants | 13 | (a) Report the numbers of individuals at each stage of the study (e.g., numbers potentially eligible, examined for eligibility, confirmed eligible, included in  the study, completing follow-up, and analysed).  (b) Give reasons for non-participation at each stage.  (c) Consider use of a flow diagram. | Results | RECORD 13.1: Describe in detail the selection of the persons included in the study (i.e., study population selection) including filtering based on data quality, data availability and linkage. The selection of included persons can be described in the text and/or by means of the study flow diagram. | Results |
| Descriptive data | 14 | (a) Give characteristics of study participants (e.g., demographic, clinical, social) and information on exposures and potential confounders.  (b) Indicate the number of participants with missing data for each variable of interest.  (c) Cohort study – summarise follow-up time (e.g., average and total amount). | Results |  |  |

**Appendix Table S1. Continued.**

|  | Item No. | STROBE items | Location in manuscript where items are reported | RECORD items | Location in manuscript where items are reported |
| --- | --- | --- | --- | --- | --- |
| Outcome data | 15 | Cohort study - Report numbers of outcome events or summary measures over time. | Results |  |  |
| Main results | 16 | (a) Give unadjusted estimates and, if applicable, confounder-adjusted estimates and their precision (e.g., 95% confidence interval). Make clear which  confounders were adjusted for and why they were included.  (b) Report category boundaries when continuous variables were categorized.  (c) If relevant, consider translating estimates of relative risk into absolute risk for a meaningful time period. | Results |  |  |
| Other analyses | 17 | Report other analyses done—e.g., analyses of subgroups and interactions, and sensitivity analyses. | Results |  |  |
| Discussion | | | | | |
| Key results | 18 | Summarise key results with reference to study objectives. | Discussion |  |  |
| Limitations | 19 | Discuss limitations of the study, taking into account sources of potential bias or imprecision. Discuss both direction and  magnitude of any potential bias. | Discussion | RECORD 19.1: Discuss the implications of using data that were not created or collected to answer the specific research question(s). Include discussion of misclassification bias, unmeasured confounding, missing data, and changing eligibility over time, as they pertain to the study being  reported. | Discussion |
| Interpretation | 20 | Give a cautious overall interpretation of results considering objectives, limitations, multiplicity of analyses, results from similar studies, and other relevant evidence. | Discussion |  |  |
| Generalisability | 21 | Discuss the generalisability (external validity) of the study results. | Discussion |  |  |
| Other information | | | | | |
| Funding | 22 | Give the source of funding and the role of the funders for the present study and, if applicable, for the original study on which the present article is based. | Funding |  |  |
| Accessibility of protocol, raw data, and progra  mming code |  | .. | Data Availability | RECORD 22.1: Authors should provide information on how to access any supplemental information such as the study protocol, raw data, or  programming code. | Data Availability |

**Appendix Table S2. STrengthening the Reporting Of Pharmacogenetic Studies (STROPS) checklist.**

| **Category** | # | Criteria | Page No |
| --- | --- | --- | --- |
| Title and abstract | 1 | Provide in the abstract an informative and balanced summary of what was done and what was found. | 1–2 |
| Introduction | | | |
| Background/rationale | 2 | Explain the scientific background and rationale for the investigation being reported. | 3 |
|  | 3 | Provide reasons for choosing the genes and SNPs genotyped. | 5 |
| Objectives | 4 | State specific objectives, including any prespecified hypotheses. | 3 |
|  | 5 | State if the study is the first report of a pharmacogenetic association, a replication  effort, or both. | 3 |
| Methods | | | |
| Study design | 6 | Present key elements of study design early in the paper. | 3 |
| Setting | 7 | Describe the setting, locations, and relevant dates, including periods of recruitment, exposure, follow-up, and data collection. | 3 |
| Participants | 8 | Give the eligibility criteria and the sources and methods of selection of participants. For a cohort study, describe methods of follow-up. For a case-control study, state whether true controls or population controls were used. Give the rationale for the choice of cases and controls. | 4 |
|  | 9 | Report the drug and regime participants were exposed to and the length of exposure. | 4 |
|  | 10 | For a matched case-control study, give matching criteria and the number of controls per case. | NA |
|  | 11 | Give information on the criteria and methods for selection of subsets of participants from a larger study, when relevant. | 4 |
|  | 12 | If other publications report results for the same patient cohort or a subset of the  patient cohort, provide information on this patient cohort overlap and references to the relevant publications. | 4 |
|  | 13 | Report disease/clinical indication of patients using a standardized ontology when  possible. | 4 |
| Variables | 14 | Clearly define all outcomes, potential confounders, and effect modifiers. Give  diagnostic criteria, if applicable. | 4 |
|  | 15 | Provide justification for choice of outcomes. | 4 |
|  | 16 | Clearly define genetic exposures (genetic variants) using a widely used nomenclature system. | 5 |
|  | 17 | Report the rs number of each genotyped SNP. | 5 |
|  | 18 | Clearly state how haplotypes or star alleles were defined. | 5 |
|  | 19 | If referring to the minor, major, wild-type, mutant, reference, risk or effect allele of a variant, state which allele this is and for which given population/cohort. | 5 |
| Data sources/ measurement | 20 | For each variable of interest, give sources of data and details of methods of  assessment (measurement). Describe comparability of assessment methods if there is more than one group. | 5 |
|  | 21 | Describe laboratory methods, including source and storage of DNA, genotyping  methods and platforms (including the allele calling algorithm used, and its version),  error rates, and call rates. State the laboratory/center where genotyping was done.  Describe comparability of laboratory methods if there is more than one group.  Specify whether genotypes were assigned using all of the data from the study  simultaneously or in smaller batches. | 5 |
|  | 22 | Describe genotype quality control methods and findings. | 5 |
|  | 23 | For quantitative outcome variables, specify if any investigation of potential bias resul ting from pharmacotherapy was undertaken. If relevant, describe the nature and magnitude of the potential bias, and explain what approach was used to deal with this. | NA |
|  | 24 | Report how adherence to treatment was assessed, and report the results of the  assessment. | None |
| Study size | 25 | Explain how the study size was arrived at, or provide details of the a priori power to detect effect sizes of varying degrees. | 5 |
| Quantitative variables | 26 | Explain how quantitative variables (confounders and effect modifiers) were handled in the analyses. If applicable, describe which groupings were chosen, and why. | 5–6 |

**Appendix Table S2. Continued.**

| **Category** | **#** | **Criteria** | **Page No^a^** |
| --- | --- | --- | --- |
| Statistical methods | 27 | Address the following: |  |
|  | (a) | Describe methods used to control for confounding. | 5–6 |
|  | (b) | Describe any methods used to examine subgroups and interactions. | 6 |
|  | (c) | Explain how missing data were addressed. | 5 |
|  | (d) | Cohort study—If applicable, explain how loss to follow-up was addressed. | 5 |
|  | (e) | Case-control study—If applicable, explain how matching of cases and controls was  addressed. | NA |
|  | (f) | Describe any sensitivity analyses. | 6 |
|  | 28 | State whether Hardy–Weinberg equilibrium was considered, and if so, how. | 5 |
|  | 29 | Describe any methods used for inferring genotypes or haplotypes. | 5 |
|  | 30 | Describe any methods used to assess or address population stratification. | 5 |
|  | 31 | Describe any methods used to assess and correct for relatedness among subjects.  Report results of assessments for relatedness. | 4 |
|  | 32 | Describe any methods used to address multiple comparisons or to control risk of  false positive results due to (a) multiple genetic variants, (b) multiple outcomes, and  (c) multiple assumptions regarding mode of inheritance. | 6 |
|  | 33 | Describe any methods used to adjust for extent of adherence in the analyses. | NA |
| Results | | | |
| Participants | 34 | Report the numbers of individuals at each stage of the study—e.g., numbers  potentially eligible, examined for eligibility, confirmed eligible, included in the  study, completing follow-up, and analyzed. | 6 |
| SNPs | 35 | Report any SNPs that were excluded from analysis, and provide reasons for these  exclusions. | NA |
| Descriptive data | 36 | Give characteristics of study participants (e.g., demographic, clinical, social,  ethnicity) and information on potential confounders. | 6 |
|  | 37 | Cohort study—Summarize follow-up time, e.g., average and/or total amount. | 7 |
|  | 38 | Where HWE tests have been undertaken, highlight SNPs that deviate from HWE. | 7 |
|  | 39 | Where population stratification is assessed, report the results. | NA |
| Outcome data | 40 | For a cross-sectional study, report all outcomes (phenotypes) investigated for each  genotype category. | NA |
|  | 41 | If a study includes more than one ethnic group, provide the summary data specified  in (40) per ethnic group. | 7 |
| Main results | 42 | Give unadjusted estimates, and if applicable, confounder-adjusted estimates and  their precision (e.g., 95% confidence intervals). Make clear which confounders were  adjusted for and why they were included. | 7 |
|  | 43 | Report category boundaries when continuous variables were categorized. | NA |
| Other analyses | 44 | Report other analyses done—e.g., analyses of subgroups and interactions, and  sensitivity analyses. | 7 |
|  | 45 | If numerous genetic exposures (genetic variants) were examined, summarize results  from all analyses undertaken. | 7 |
|  | 46 | If detailed results are available elsewhere, i.e., in supplementary materials, state how  they can be accessed. | 7 |
| Discussion | | | |
| Key results | 47 | Summarise key results with reference to study objectives. | 8 |
| Limitations | 48 | Discuss limitations of the study, taking into account sources of potential bias or imprecision. Discuss both direction and magnitude of any potential bias. | 8–9 |
| Interpretation | 49 | Give a cautious overall interpretation of results considering objectives, limitations, multiplicity of analyses, results from similar studies, and other relevant evidence. | 8 |
| Generalizability | 50 | Discuss the generalisability (external validity) of the study results. | 8–9 |
| Other information | | | |
| Study registration | 51 | State whether the study has been registered. If the study has been registered, provide details of the registry. | NA |
| Ethical approval | 52 | Report whether ethical approval was obtained for the collection of genetic data. | 3 |
| Funding | 53 | Give the source of funding and the role of the funders for the present study and, if applicable, for the original study on which the present article is based. | 9 |
| Databases | 54 | State whether databases for the analyzed data are or will become publicly available,  and if so, how they can be accessed. | 9 |

HWE, Hardy–Weinberg equilibrium; NA, not applicable; rs, reference SNP cluster ID; SNP, single nucleotide polymorphism.

**Appendix Table S3. ACMI medications list**

| **Drug** | **Score** | **Drug** | **Score** |
| --- | --- | --- | --- |
| desloratadine | 0.1 | metoprolol | 1.2 |
| loratadine | 0.2 | oxybutynin | 1.2 |
| dipyridamole | 0.4 | umeclidinium | 1.2 |
| alverine | 0.5 | temazepam | 1.2 |
| colchicine | 0.6 | hyoscine (scopolamine) | 1.2 |
| betamethasone | 0.6 | hydrocortisone | 1.2 |
| captopril | 0.6 | tiotropium | 1.2 |
| clonidine | 0.6 | lorazepam | 1.3 |
| bendroflumethiazide | 0.7 | codeine | 1.3 |
| warfarin | 0.7 | citalopram | 1.3 |
| indapamide | 0.7 | baclofen | 1.3 |
| chlorpromazine | 0.7 | diazepam | 1.3 |
| hydroxyzine | 0.7 | mirtazapine | 1.3 |
| dosulepin | 0.8 | buprenorphine | 1.3 |
| imipramine | 0.8 | quetiapine | 1.3 |
| hydrochlorothiazide | 0.8 | trospium | 1.3 |
| solifenacin | 0.8 | risperidone | 1.3 |
| glycopyrronium | 0.9 | ranitidine | 1.3 |
| doxazosin | 0.9 | venlafaxine | 1.3 |
| dihydrocodeine | 0.9 | cinnarizine | 1.3 |
| promethazine | 0.9 | darifenacin | 1.3 |
| atenolol | 0.9 | bumetanide | 1.4 |
| cetirizine | 0.9 | tramadol | 1.4 |
| clomipramine | 0.9 | cimetidine | 1.4 |
| loperamide | 0.9 | fentanyl | 1.4 |
| amitriptyline | 1 | fluoxetine | 1.4 |
| mebeverine | 1 | amisulpride | 1.4 |
| nortriptyline | 1 | indoramin | 1.4 |
| prochlorperazine | 1 | morphine | 1.5 |
| theophylline | 1 | haloperidol | 1.5 |
| tolterodine | 1 | hydralazine | 1.5 |
| trazodone | 1 | aripiprazole | 1.5 |
| fexofenadine | 1 | oxycodone | 1.5 |
| isosorbide | 1 | ipratropium | 1.5 |
| prednisolone | 1.1 | olanzapine | 1.5 |
| flupentixol | 1.1 | procyclidine | 1.5 |
| dexamethasone | 1.1 | carbamazepine | 1.5 |
| digoxin | 1.1 | escitalopram | 1.6 |
| nefopam | 1.1 | fesoterodine | 1.6 |
| nifedipine | 1.1 | cyclizine | 1.7 |
| clonazepam | 1.1 | trihexyphenidyl | 1.7 |
| furosemide | 1.1 | midazolam | 1.8 |
| chlorphenamine (chlorpheniramine) | 1.2 | atropine | 1.9 |
| paroxetine | 1.2 | amantadine | 2.3 |

**Appendix Table S4. Acetylcholine signalling pathway genes[10-13]**

| **Pathway feature** | **Genes involved** |
| --- | --- |
| Synthesis | ***SLC5A7*** (solute carrier family 5 member 7): transporting choline into the presynaptic nerve  ***CHAT*** (choline O-acetyltransferase): synthesizing acetylcholine (Ach) from choline and acetyl-CoA. |
| Storage | ***SLC18A3*** (Solute carrier family 18, member 3): transporting Ach into storage vesicles |
| Release | ***VAMP2*** (vesicle associated membrane protein 2): interacts with calcium to trigger release of Ach into the synapse. |
| Termination of action | ***ACHE*** (acetylcholinesterase): metabolises ACh |
| Receptor effects | ***CHRM1-5*** (cholinergic receptor muscarinic 1-5)  ***CHRNA1-7***, ***9-10*** (cholinergic receptor nicotinic alpha 1-7, 9-10 subunit)  ***CHRNB1-4*** (cholinergic receptor nicotinic beta 1-4 subunit)  ***CHRND*** (cholinergic receptor nicotinic delta subunit)  ***CHRNE*** (cholinergic receptor nicotinic epsilon subunit)  ***CHRNG*** (cholinergic receptor nicotinic gamma subunit) |
| Signalling mechanism | *Cholinoceptors act either through G protein-linked (muscarinic) or ion channel (nicotinic) signalling mechanisms.*  ***GRK2/5*** (G protein-coupled receptor kinase 2/5)  ***RGS1-14, 16-21*** (regulator of G protein signaling 1-14, 16-21)  ***GNAI1/2*** (G protein subunit alpha i1/2)  ***GNA11/4*** (G protein subunit alpha 11)  ***GNAQ*** (G protein subunit alpha q) |
| Neuromuscular junction related | ***AGRN*** (agrin)  ***DOK7*** (docking protein 7)  ***LRP4*** (LDL receptor related protein 4)  ***MUSK*** (muscle associated receptor tyrosine kinase)  ***RAPSN*** (receptor associated protein of the synapse) |
| Potassium voltage-gated channel (KCN) genes. | Kv1 (KCNA), Shaker-related family: ***KCNA1, KCNA2, KCNA3, KCNA4, KCNA5, KCNA6, KCNA7, KCNA10***  Kv2 (KCNB), Shab-related family: ***KCNB1, KCNB2***  Kv3 (KCNC), Shaw-related family: ***KCNC1, KCNC2, KCNC3, KCNC4***  Kv4 (KCND), Shal-related family: ***KCND2, KCND3***  Kv7 (KCNQ), KVLQT and KQT2 family: ***KCNQ1, KCNQ2, KCNQ3, KCNQ4, KCNQ5***  Kv10-Kv12 (KCNH), eag (10), erg (11), elk (12) family: ***KCNH1, KCNH2, KCNH3, KCNH5, KCNH6, KCNH7, KCNH8***  Modifiers: ***KCNS1, KCNS2, KCNS3, KCNV1, KCNV2, KCNF1, KCNG1, KCNG4*** |

**Appendix Table S5. Characteristics of UK Biobank participants.**

|  | **Censored (N=123417)** | **With Dementia (N=1843)** | **Overall (N=125260)** |
| --- | --- | --- | --- |
| **Age (years)** |  |  |  |
| Mean (SD) | 54.4 (5.88) | 57.2 (3.88) | 54.5 (5.87) |
| Median [Min, Max] | 54.1 [42.8, 77.3] | 57.6 [43.8, 71.7] | 54.2 [42.8, 77.3] |
| **Sex** |  |  |  |
| Female | 66715 (54.1%) | 875 (47.5%) | 67590 (54.0%) |
| Male | 56702 (45.9%) | 968 (52.5%) | 57670 (46.0%) |
| **Race** |  |  |  |
| White | 119226 (96.6%) | 1802 (97.8%) | 121028 (96.6%) |
| Asian | 2036 (1.6%) | 16 (0.9%) | 2052 (1.6%) |
| Black | 716 (0.6%) | 8 (0.4%) | 724 (0.6%) |
| Mixed/other/unknown | 1439 (1.2%) | 17 (0.9%) | 1456 (1.2%) |
| **Registration after 1st Jan 1999** |  |  |  |
| No | 82344 (66.7%) | 1466 (79.5%) | 83810 (66.9%) |
| Yes | 41073 (33.3%) | 377 (20.5%) | 41450 (33.1%) |
| **Data provider** |  |  |  |
| England (TPP) | 87537 (70.9%) | 875 (47.5%) | 88412 (70.6%) |
| England (Vision) | 10251 (8.3%) | 281 (15.2%) | 10532 (8.4%) |
| Scotland | 13305 (10.8%) | 289 (15.7%) | 13594 (10.9%) |
| Wales | 12324 (10.0%) | 398 (21.6%) | 12722 (10.2%) |
| **Genotyping array** |  |  |  |
| Axiom | 110191 (89.3%) | 1637 (88.8%) | 111828 (89.3%) |
| BiLEVE | 13226 (10.7%) | 206 (11.2%) | 13432 (10.7%) |
| ***APOE* carrier** |  |  |  |
| *ε2* | 16098 (13.0%) | 180 (9.8%) | 16278 (13.0%) |
| *ε3* | 76236 (61.8%) | 918 (49.8%) | 77154 (61.6%) |
| *ε4* | 31083 (25.2%) | 745 (40.4%) | 31828 (25.4%) |
| **Prior depression** |  |  |  |
| No | 118082 (95.7%) | 1746 (94.7%) | 119828 (95.7%) |
| Yes | 5335 (4.3%) | 97 (5.3%) | 5432 (4.3%) |
| **Prior diabetes** |  |  |  |
| No | 117729 (95.4%) | 1782 (96.7%) | 119511 (95.4%) |
| Yes | 5688 (4.6%) | 61 (3.3%) | 5749 (4.6%) |
| **Prior hypercholesterolemia** |  |  |  |
| No | 113708 (92.1%) | 1770 (96.0%) | 115478 (92.2%) |
| Yes | 9709 (7.9%) | 73 (4.0%) | 9782 (7.8%) |
| **Prior hypertension** |  |  |  |
| No | 103321 (83.7%) | 1661 (90.1%) | 104982 (83.8%) |
| Yes | 20096 (16.3%) | 182 (9.9%) | 20278 (16.2%) |
| **Prior stroke** |  |  |  |
| No | 120956 (98.0%) | 1818 (98.6%) | 122774 (98.0%) |
| Yes | 2461 (2.0%) | 25 (1.4%) | 2486 (2.0%) |
| **On at least one ACMI-listed drug** |  |  |  |
| No | 93061 (75.4%) | 1420 (77.0%) | 94481 (75.4%) |
| Yes | 30356 (24.6%) | 423 (23.0%) | 30779 (24.6%) |
| **On at least one Duran-listed drug** |  |  |  |
| No | 90536 (73.4%) | 1413 (76.7%) | 91949 (73.4%) |
| Yes | 32881 (26.6%) | 430 (23.3%) | 33311 (26.6%) |
| **ACMI (count-based scale)** |  |  |  |
| Mean (SD) | 0.406 (0.885) | 0.391 (0.868) | 0.406 (0.885) |
| Median [Min, Max] | 0 [0, 15.0] | 0 [0, 6.00] | 0 [0, 15.0] |
| **Duran (count-based scale)** |  |  |  |
| Mean (SD) | 0.445 (0.935) | 0.396 (0.880) | 0.444 (0.934) |
| Median [Min, Max] | 0 [0, 13.0] | 0 [0, 7.00] | 0 [0, 13.0] |
| **ACMI (value-based scale)** |  |  |  |
| Mean (SD) | 0.414 (0.941) | 0.394 (0.907) | 0.414 (0.940) |
| Median [Min, Max] | 0 [0, 14.8] | 0 [0, 7.10] | 0 [0, 14.8] |
| **Duran (value-based scale)** |  |  |  |
| Mean (SD) | 0.377 (0.868) | 0.341 (0.824) | 0.376 (0.867) |
| Median [Min, Max] | 0 [0, 17.0] | 0 [0, 8.00] | 0 [0, 17.0] |
| **Follow-up time, dementia (years)** |  |  |  |
| Mean (SD) | 13.9 (4.16) | 8.84 (4.30) | 13.8 (4.21) |
| Median [Min, Max] | 16.4 [1.00, 17.7] | 8.92 [1.00, 17.3] | 16.4 [1.00, 17.7] |
| **Death status** |  |  |  |
| Censored | 119901 (97.2%) | 1728 (93.8%) | 121629 (97.1%) |
| Died | 3516 (2.8%) | 115 (6.2%) | 3631 (2.9%) |
| **Follow-up time, death (years)** |  |  |  |
| Mean (SD) | 13.9 (4.16) | 15.1 (2.67) | 13.9 (4.15) |
| Median [Min, Max] | 16.4 [1.00, 17.7] | 16.4 [1.32, 17.7] | 16.4 [1.00, 17.7] |

**Abbreviations:** ACMI = Anticholinergic Medication Index, APOE = Apolipoprotein E, Axiom = the Applied Biosystems UK Biobank Axiom Array, BiLEVE = the Applied Biosystems UK Biobank Lung Exome Variant Evaluation Axiom Array by Affymetrix, Max = Maximum, Min = Minimum, N = number of participants, SD = standard deviation, TPP = The Phoenix Partnership.

**Appendix Table S6. Characteristics of the All of Us program participants.**

|  | **Censored (N=90483)** | **With Dementia (N=1564)** | **Overall (N=92047)** |
| --- | --- | --- | --- |
| **Age (years)** |  |  |  |
| Mean (SD) | 59.7 (9.68) | 61.7 (9.70) | 59.7 (9.69) |
| Median [Min, Max] | 59.7 [37.5, 115] | 61.4 [39.5, 94.4] | 59.7 [37.5, 115] |
| **Sex** |  |  |  |
| Female | 50208 (55.5%) | 863 (55.2%) | 51071 (55.5%) |
| Male | 40275 (44.5%) | 701 (44.8%) | 40976 (44.5%) |
| **Race** |  |  |  |
| White | 60260 (66.6%) | 1045 (66.8%) | 61305 (66.6%) |
| Asian | 1686 (1.9%) | 26 (1.7%) | 1712 (1.9%) |
| Black | 15289 (16.9%) | 202 (12.9%) | 15491 (16.8%) |
| Mixed/other/unknown | 13248 (14.6%) | 291 (18.6%) | 13539 (14.7%) |
| **First recorded electronic health record after 1st Jan 1999** |  |  |  |
| No | 12280 (13.6%) | 412 (26.3%) | 12692 (13.8%) |
| Yes | 78203 (86.4%) | 1152 (73.7%) | 79355 (86.2%) |
| ***APOE* carrier** |  |  |  |
| *ε2* | 11859 (13.1%) | 182 (11.6%) | 12041 (13.1%) |
| *ε3* | 56968 (63.0%) | 893 (57.1%) | 57861 (62.9%) |
| *ε4* | 21656 (23.9%) | 489 (31.3%) | 22145 (24.1%) |
| **Prior depression** |  |  |  |
| No | 85863 (94.9%) | 1385 (88.6%) | 87248 (94.8%) |
| Yes | 4620 (5.1%) | 179 (11.4%) | 4799 (5.2%) |
| **Prior diabetes** |  |  |  |
| No | 84290 (93.2%) | 1362 (87.1%) | 85652 (93.1%) |
| Yes | 6193 (6.8%) | 202 (12.9%) | 6395 (6.9%) |
| **Prior hypercholesterolemia** |  |  |  |
| No | 86273 (95.3%) | 1404 (89.8%) | 87677 (95.3%) |
| Yes | 4210 (4.7%) | 160 (10.2%) | 4370 (4.7%) |
| **Prior hypertension** |  |  |  |
| No | 74186 (82.0%) | 1067 (68.2%) | 75253 (81.8%) |
| Yes | 16297 (18.0%) | 497 (31.8%) | 16794 (18.2%) |
| **Prior stroke** |  |  |  |
| No | 90465 (100.0%) | 1564 (100%) | 92029 (100.0%) |
| Yes | 18 (0.0%) | 0 (0%) | 18 (0.0%) |
| **On at least one ACMI-listed drug** |  |  |  |
| No | 70389 (77.8%) | 1115 (71.3%) | 71504 (77.7%) |
| Yes | 20094 (22.2%) | 449 (28.7%) | 20543 (22.3%) |
| **ACMI (count-based scale)** |  |  |  |
| Mean (SD) | 0.634 (1.63) | 0.895 (1.93) | 0.638 (1.64) |
| Median [Min, Max] | 0 [0, 22.0] | 0 [0, 14.0] | 0 [0, 22.0] |
| **Follow-up time, dementia (years)** |  |  |  |
| Mean (SD) | 10.8 (7.30) | 11.4 (6.33) | 10.8 (7.28) |
| Median [Min, Max] | 9.24 [1.00, 22.5] | 11.0 [1.01, 22.5] | 9.27 [1.00, 22.5] |
| **Death status** |  |  |  |
| Censored | 89306 (98.7%) | 1467 (93.8%) | 90773 (98.6%) |
| Died | 1177 (1.3%) | 97 (6.2%) | 1274 (1.4%) |
| **Follow-up time, death (years)** |  |  |  |
| Mean (SD) | 10.8 (7.29) | 15.1 (6.38) | 10.8 (7.30) |
| Median [Min, Max] | 9.22 [1.00, 22.5] | 15.9 [1.91, 22.5] | 9.31 [1.00, 22.5] |

**Abbreviations:** ACMI = Anticholinergic Medication Index, APOE = Apolipoprotein E, Max = Maximum, Min = Minimum, N = number of participants, SD = standard deviation.

**Supplementary Figures**

**All dementia cases (n = 6,736)** **Dementia cases by** **31st May 2016 (n = 6,486)**


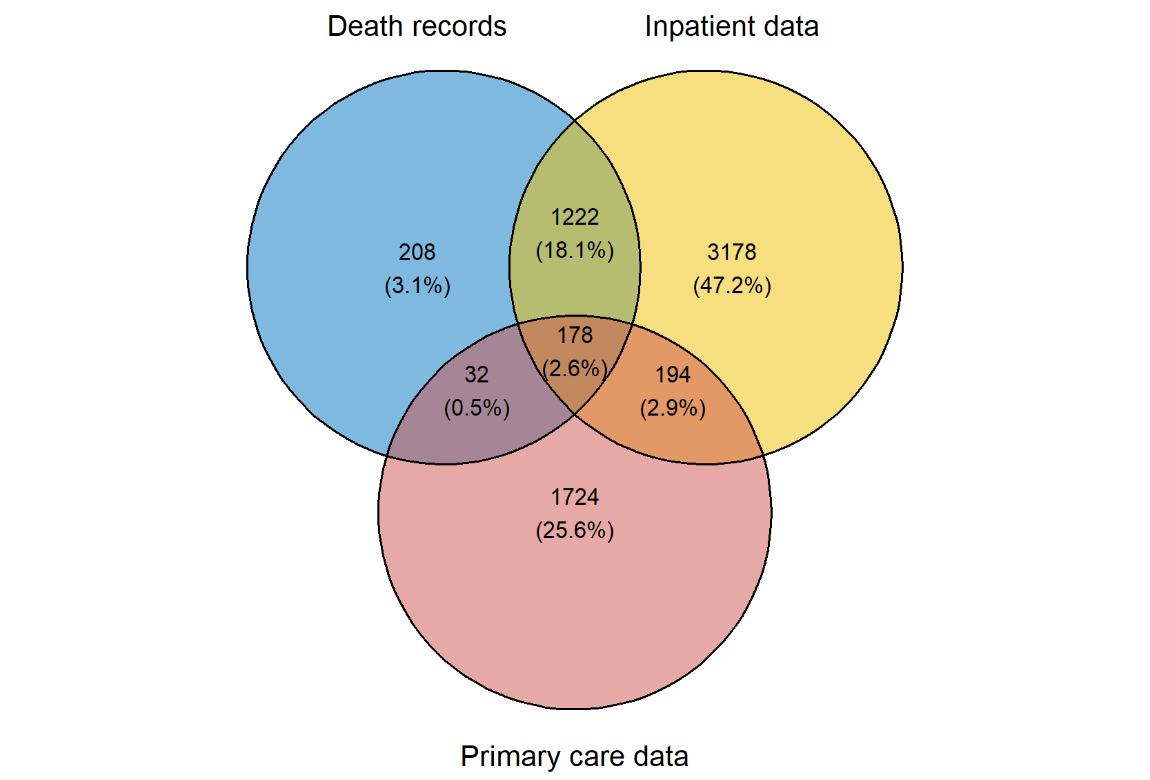

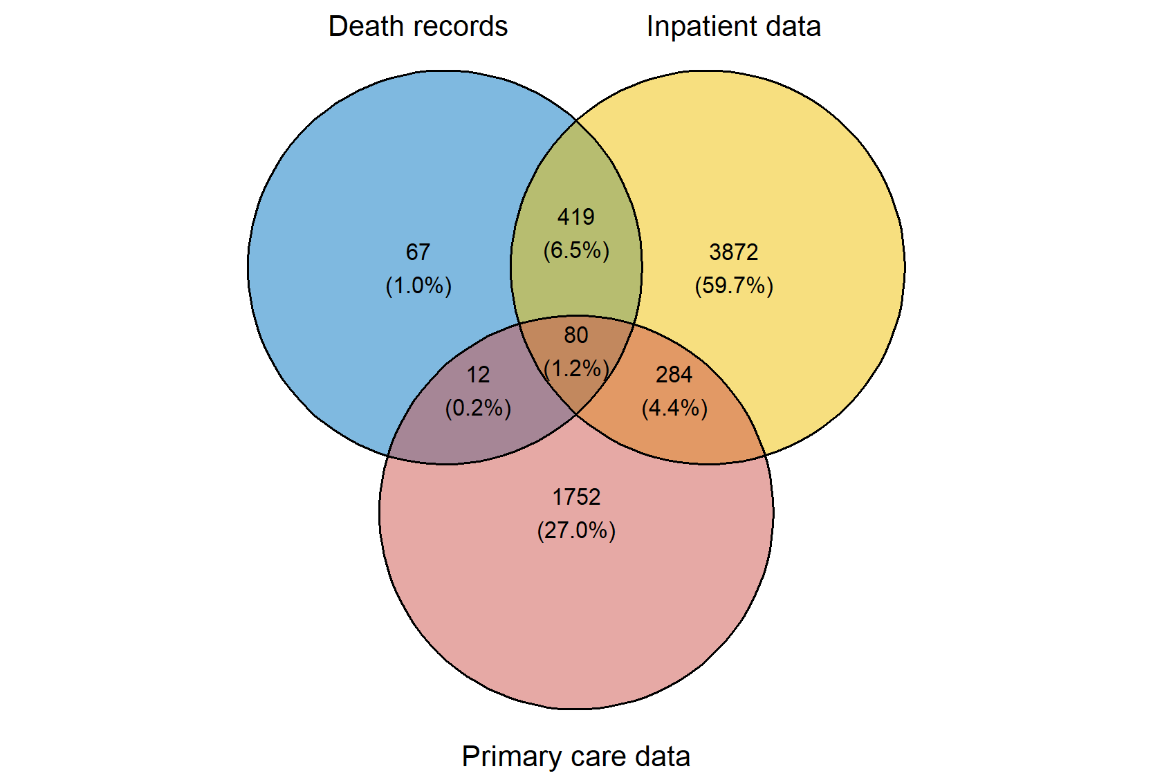


**Appendix Figure S1. Distribution of dementia cases among the different data sources in UK Biobank participants with linked healthcare records (n =** **219,141).** Most cases are sourced from inpatient data, with a censor date of 31^st^ October 2022. Compared to using a censor date of 31^st^ May 2016 (applicable to the primary care data), there would be 250 more cases when follow-up ends on 31^st^ October 2022. However, for each additional three cases, about one dementia case (74%:26%) could potentially be misclassified as not having dementia. The sample size in this analysis (219,141) is slightly greater than that in Figure 1 (215,973) as genotype data wasn’t considered.

**
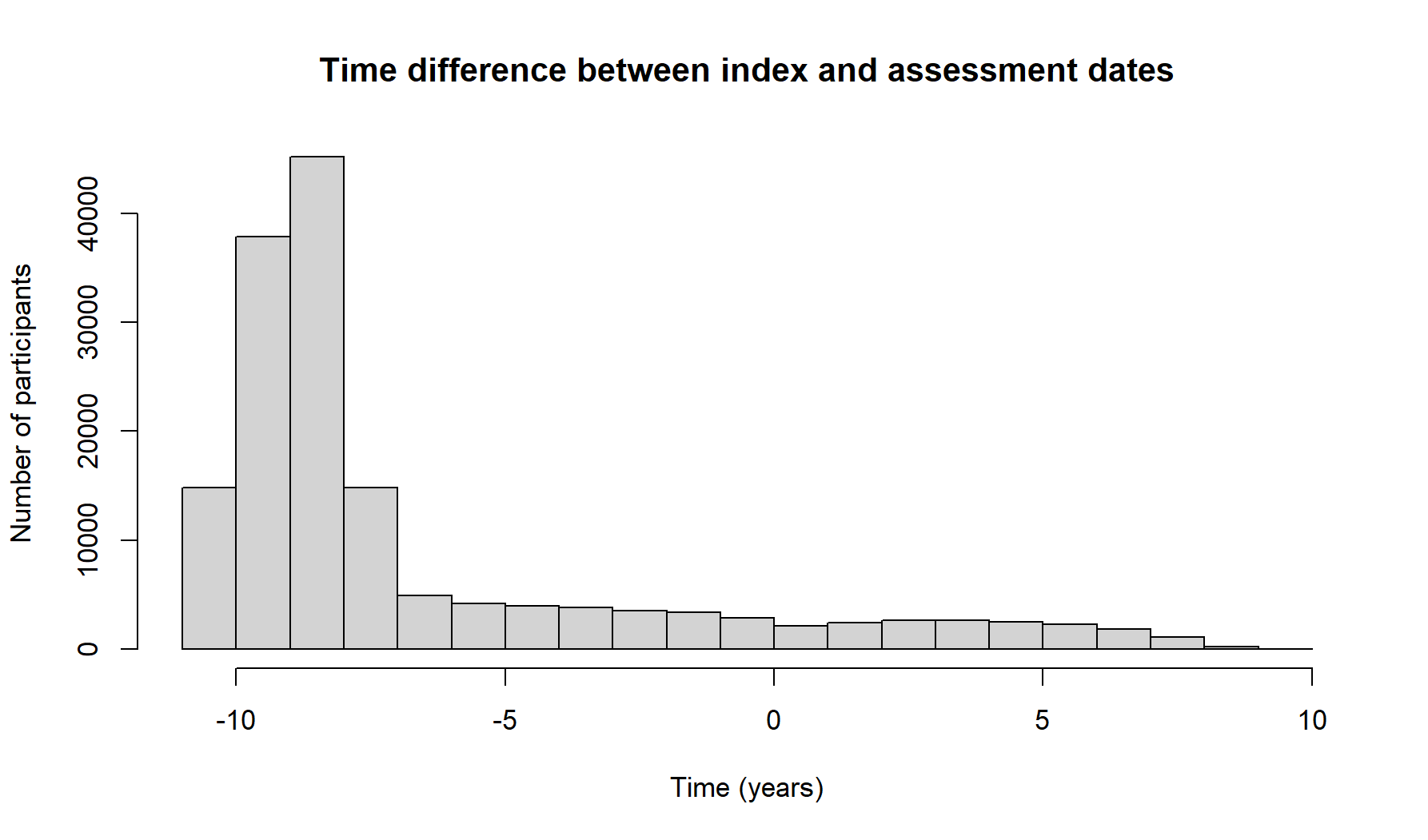
**

**Appendix Figure S2. Time difference between the index date (start of follow-up) and the date of attending UK Biobank assessment centre.**

Dementia

Start of follow-up

Death

**Competing risk**

Dementia

Start of follow-up

Death

**Multi-state 1**

Dementia

Start of follow-up

Death without dementia

**Multi-state 2**

Death after dementia

**Appendix Figure S3. Cox-proportional hazards models[14] tested during analysis.** Due to computational efficiency, the competing risk model was used as the primary method, with the two being used in sensitivity analysis.


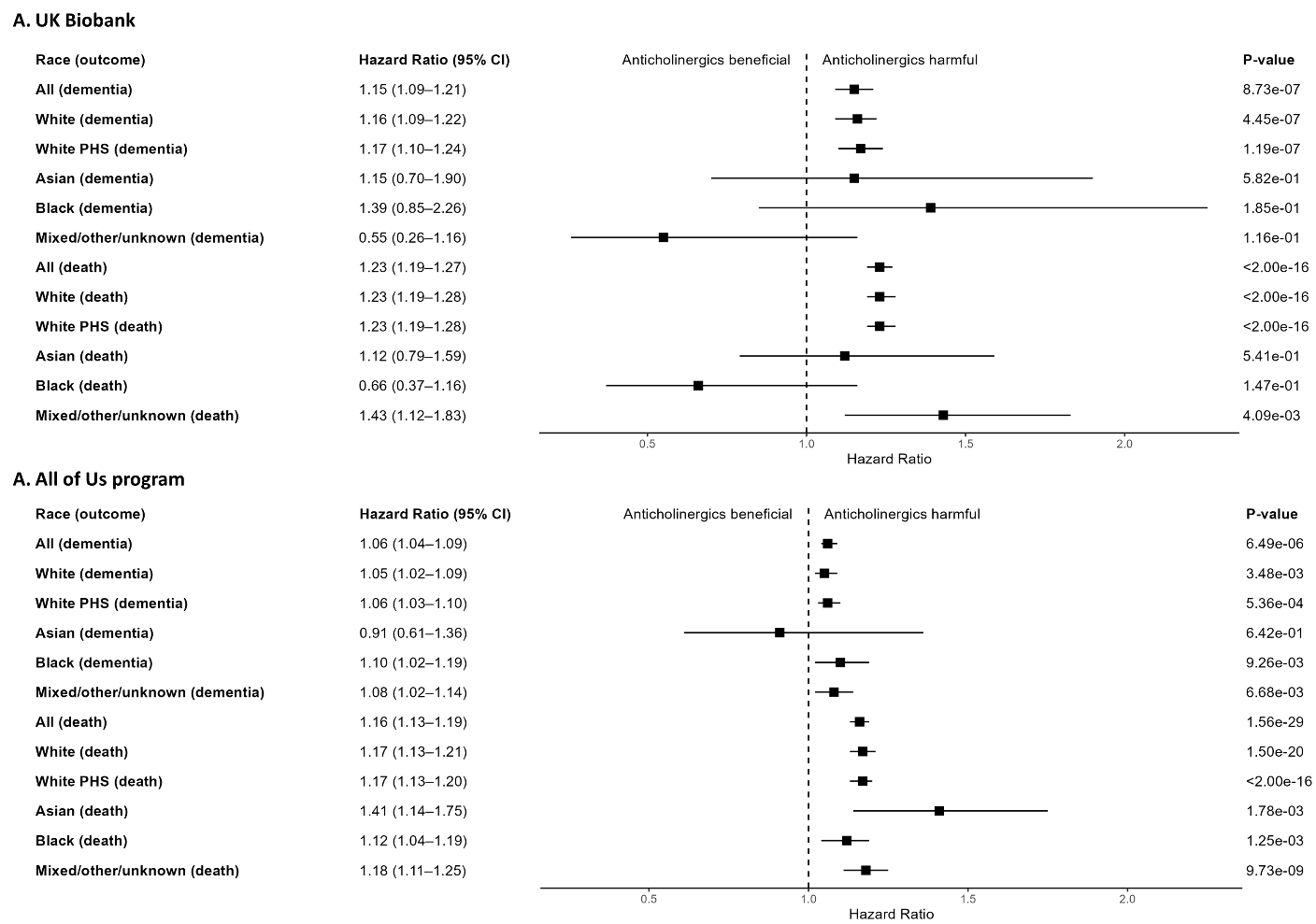


**Appendix Figure S4. ACMI’s prediction of dementia and death in the UK Biobank (panel A) and All of Us program (panel B), with race stratification.** All models were adjusted for age at index date, sex, data provider, ‘registration before 1st January 1999’ status, race (for non-stratified analyses), genotyping array, apolipoprotein A carrier status, prior comorbidities (depression, diabetes, hypercholesterolemia, hypertension, and stroke) and the first two principal components of genetic ancestry. ACMI = Anticholinergic Medication Index, CI = confidence interval. White PHS = White participants included in the polygenic hazard score analysis and include UK Biobank participants with White British ancestry and All of Us participants with genetically predicted European ancestry.


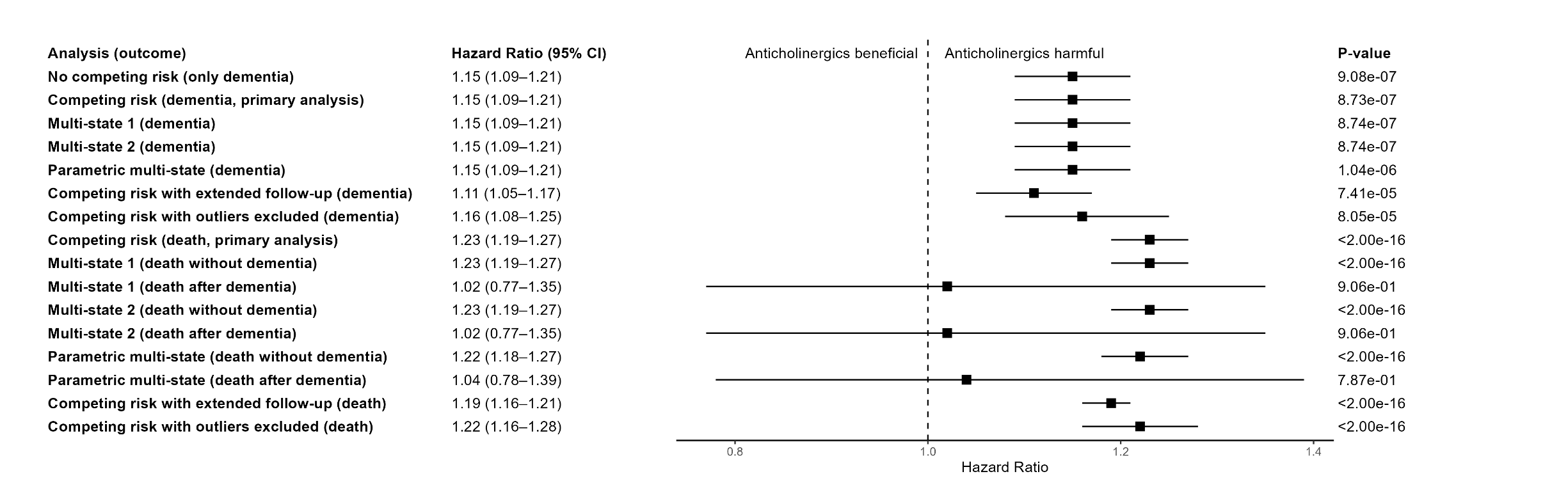


**Appendix Figure S5. Sensitivity analysis of ACMI’s prediction of dementia and death in the UK Biobank.** The multi-state 1/2 are cox-proportional hazard models in which patients with dementia could progress to death, with deaths without dementia and deaths after dementia being treated equivalently in multi-state 1, and being distinguished in multi-state 2. All models were adjusted for age at index date, sex, data provider, ‘registration before 1st January 1999’ status, race, genotyping array, apolipoprotein A carrier status, prior comorbidities (depression, diabetes, hypercholesterolemia, hypertension, and stroke) and the first two principal components of genetic ancestry. ACMI = Anticholinergic Medication Index, CI = confidence interval.

**
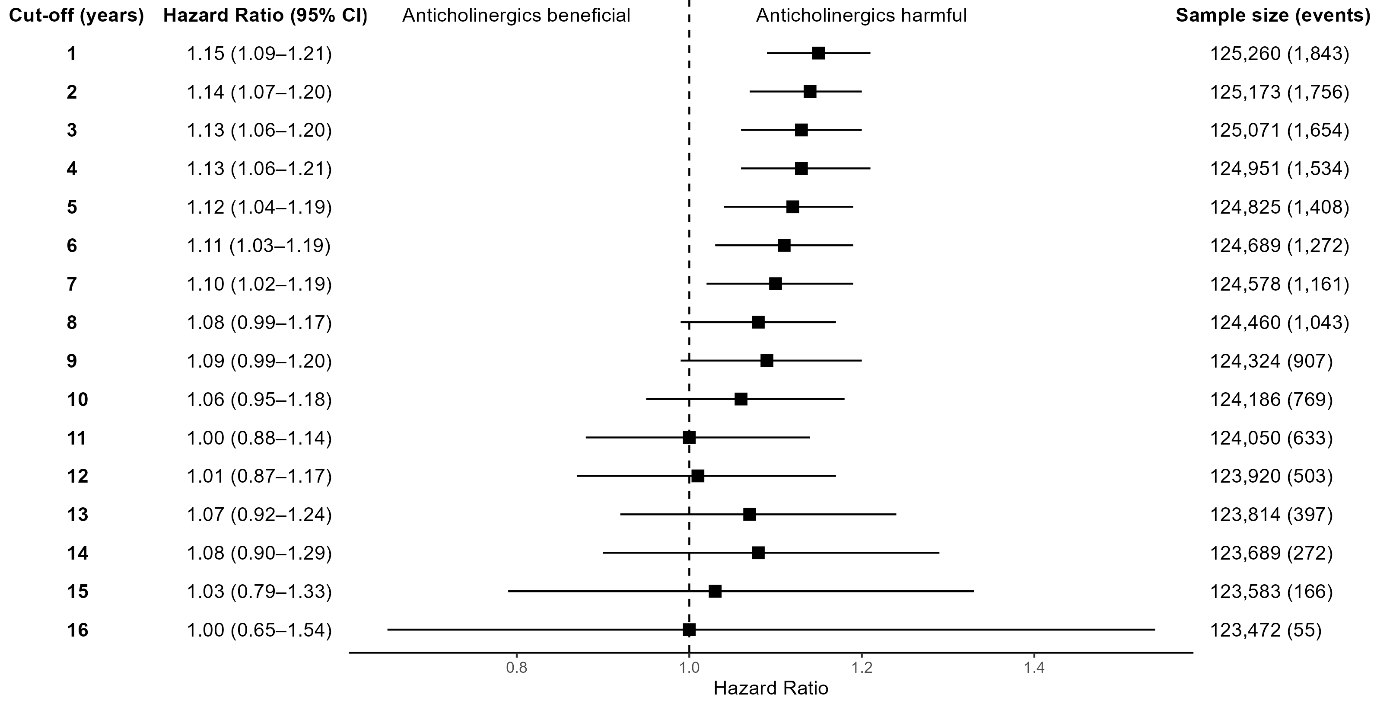
**

**Appendix Figure S6. Relationship between Anticholinergic Burden (ACMI count-based score) and dementia, considering various exclusion periods for the time of dementia diagnosis.** Participants diagnosed with dementia within a given timeframe (one year for the primary analysis) were excluded from diagnosis. As this cut-off increases, the sample size and number of dementia cases (events in the plot) decreases resulting in less precise results (wider confidence intervals). ACMI = Anticholinergic Medication Index, CI = confidence interval.

**
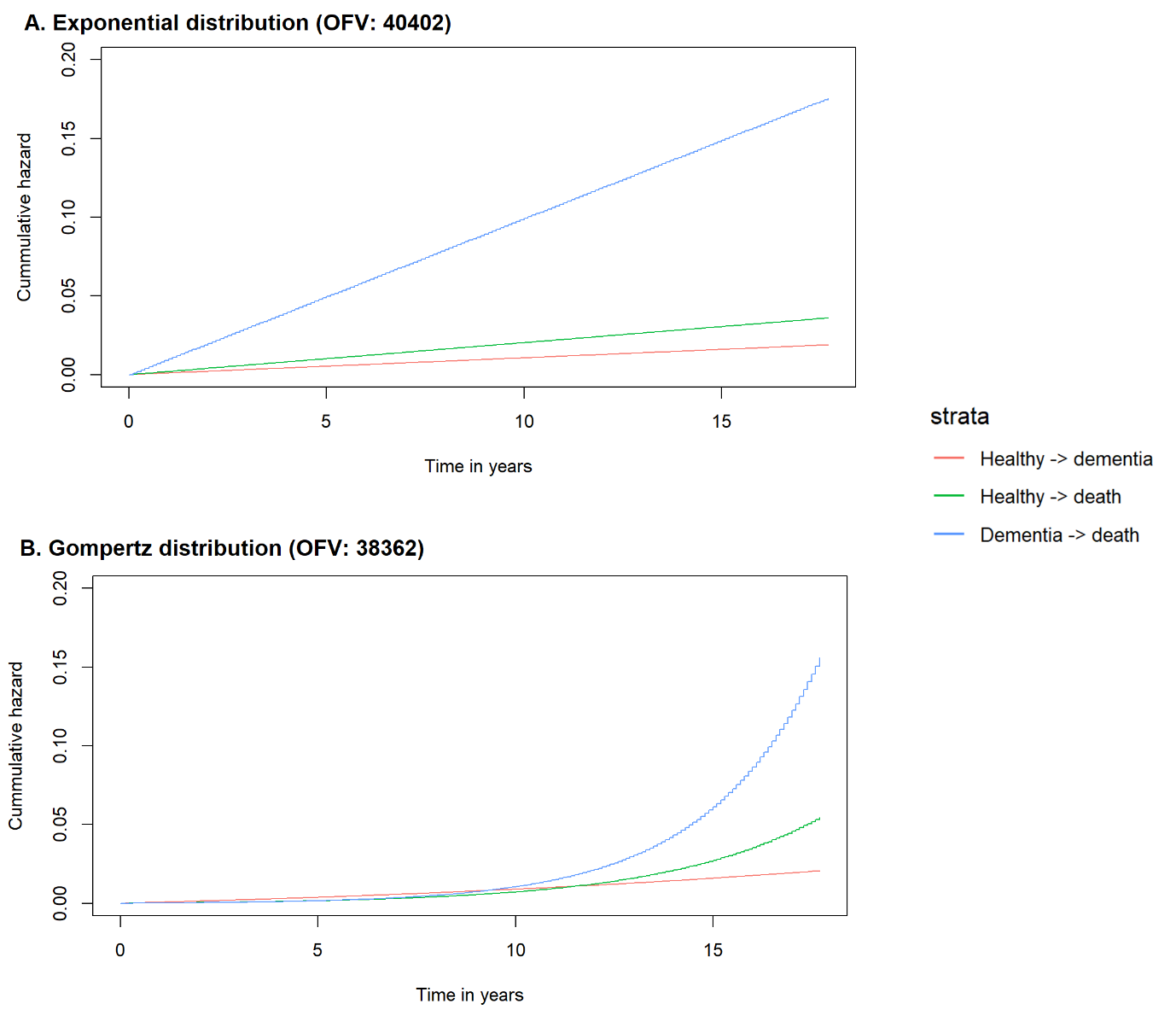
**

**Appendix Figure S7. Cumulative hazard plots for** **exponential (panel A) and Gompertz (panel B) distributions (no covariates added).** To perform parametric transition-specific modelling, a distribution needed to be specified. We utilized the R flexsurv package,[15] which offers 10 built-in distributions (Generalized gamma (stable), Generalized gamma (original), Generalized F (stable), Generalized F (original), Weibull, Gamma, Exponential, Log-logistic, Log-normal, and Gompertz). Regarding covariate addition (which was planned), only the exponential and Gompertz distributions model covariates on the location (e.g. rate) parameter using the proportional hazards models (the rest use an accelerated failure time model),[15] and so they were selected since the primary analyses employed a proportional hazards model. The Gompertz distribution was ultimately selected due to better fit, represented by a lower objective function value (OFV), where the OFV equals the negative of the logarithm of the model’s likelihood.


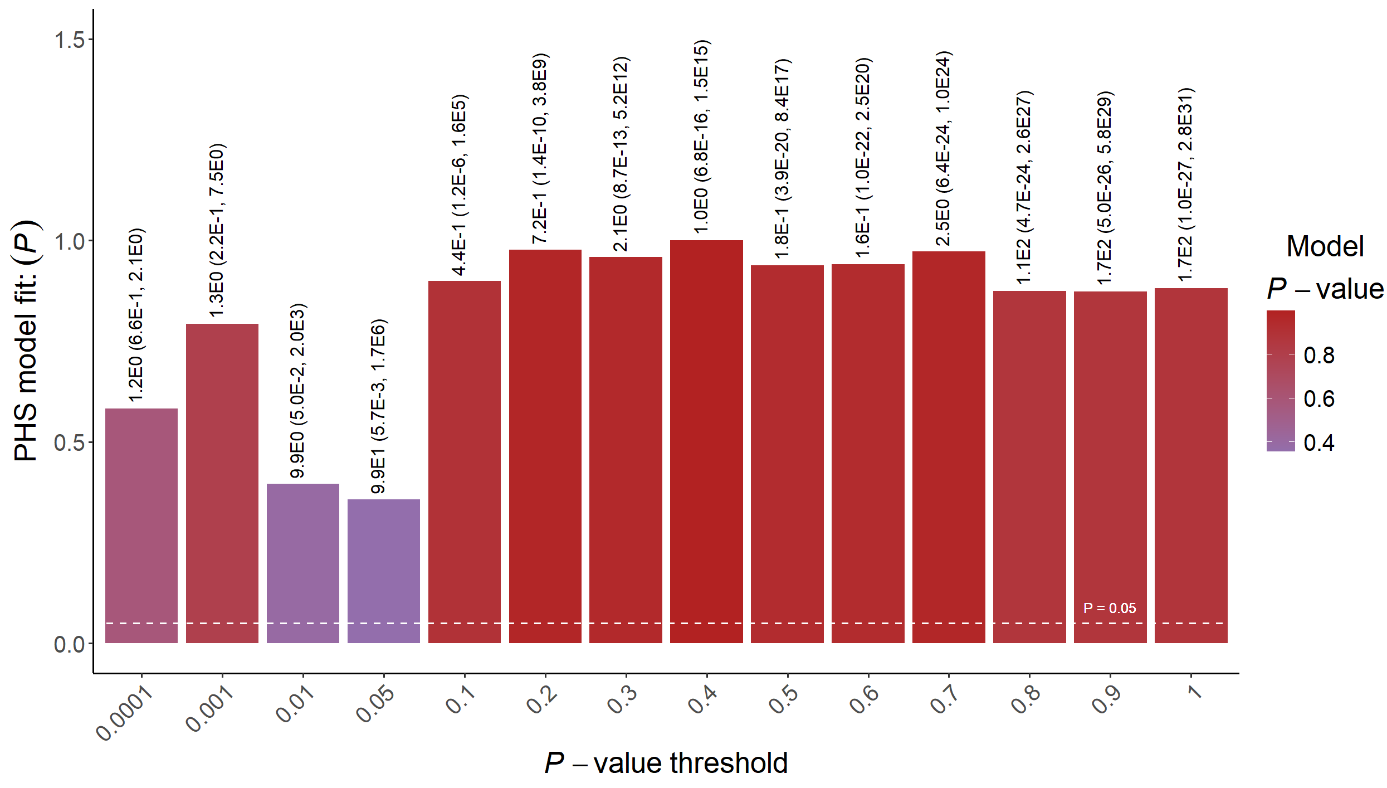
 **Appendix Figure S8. Polygenic hazard score (PHS) results corresponding to a range of P-value thresholds obtained by application of the clumping and thresholding method in PLINK.** Values at the top of the bars represent dementia hazard ratios and associated 95% confidence intervals.

**Supplementary References**

1. Barrett JC, Fry B, Maller J, Daly MJ. Haploview: analysis and visualization of LD and haplotype maps. Bioinformatics. 2005 Jan 15;21(2):263-5.

2. Benjamini Y, Hochberg Y. Controlling the False Discovery Rate: A Practical and Powerful Approach to Multiple Testing. Journal of the Royal Statistical Society: Series B (Methodological). 1995;57(1):289-300.

3. Chang CC, Chow CC, Tellier LC, Vattikuti S, Purcell SM, Lee JJ. Second-generation PLINK: rising to the challenge of larger and richer datasets. Gigascience. 2015;4:7.

4. Choi SW, Mak TS, O'Reilly PF. Tutorial: a guide to performing polygenic risk score analyses. Nat Protoc. 2020 Sep;15(9):2759-72.

5. Kent WJ, Sugnet CW, Furey TS, Roskin KM, Pringle TH, Zahler AM, et al. The human genome browser at UCSC. Genome Res. 2002 Jun;12(6):996-1006.

6. Python Software Foundation. Python (version 3.10) [Software]. 2021.

7. Hail Team. Hail (version 0.2.126) [Software]. 2023.

8. Apache Software Foundation. Apache Spark (version 3.3.0) [Software]. 2023.

9. R Core Team. R: A language and environment for statistical computing. Vienna, Austria: R Foundation for Statistical Computing; 2022.

10. Alexander SPH, Kelly E, Mathie A, Peters JA, Veale EL, Armstrong JF, et al. THE CONCISE GUIDE TO PHARMACOLOGY 2019/20: Introduction and Other Protein Targets. Br J Pharmacol. 2019 Dec;176 Suppl 1(Suppl 1):S1-S20.

11. Attali B, Chandy K, Giese M, Grissmer S, Gutman G, Jan L, et al. Voltage-gated potassium channels (Kv) in GtoPdb v.2023.1. IUPHAR/BPS Guide to Pharmacology. 2023;2023(1).

12. B.G.(Ed.) K. Basic and Clinical Pharmacology 14th Edition. New York: McGraw-Hill Education; 2018.

13. Petri V, Shimoyama M, Hayman GT, Smith JR, Tutaj M, de Pons J, et al. The Rat Genome Database pathway portal. Database (Oxford). 2011;2011:bar010.

14. Therneau T, Crowson C, Atkinson E. Multi-state models and competing risks. The Comprehensive R Archive Network: The Comprehensive R Archive Network; 2024.

15. Jackson C. flexsurv: A Platform for Parametric Survival Modeling in R. Journal of Statistical Software. 2016;80(8):1-33.
